# Umbilical metastases: current viewpoint

**DOI:** 10.1186/1477-7819-3-13

**Published:** 2005-02-21

**Authors:** Raimondo Gabriele, Marco Conte, Federico Egidi, Mario Borghese

**Affiliations:** 1 Department of Surgery, "P.Valdoni" – University of Rome "La Sapienza", Rome, Italy

## Abstract

**Background:**

Umbilical metastases from a malignant neoplasm, also termed Sister Mary Joseph's nodule, are not commonly reported in the English literature, and they have usually been considered as a sign of a poor prognosis for the patient. The present article reports on the current view point on umbilical metastasis besides discussing the epidemiology, clinical presentation, pathophysiology and treatment.

**Method:**

A search of Pubmed was carried out using the term 'umblic*' and 'metastases' or metastasis' revealed no references. Another search was made using the term "Sister Joseph's nodule" or sister Joseph nodule" that revealed 99 references. Of these there were 14 review articles, however when the search was limited to English language it yielded only 20 articles. Articles selected from these form the basis of this report along with cross references.

**Results:**

The primary lesions usually arise from gastrointestinal or genitourinary tract malignancies and may be the presenting symptom or sign of a primary tumour in an unknown site.

**Conclusion:**

A careful evaluation of all umbilical lesions, including an early biopsy if appropriate, is recommended. Recent studies suggest an aggressive surgical approach combined with chemotherapy for such patients may improve survival.

## Background

Cutaneous metastases localised to the umbilicus are named "Sister Mary Joseph's nodules". In 1949 Sir Hamilton Bailey initially used this eponym in his book "Physical Signs in Clinical Surgery" to describe umbilical metastases, in honour of Sister Mary Joseph, the superintendent nurse and surgical assistant of Dr. William Mayo at St. Mary's Hospital in Rochester (presently the Mayo Clinic in Minnesota, USA). Sister Mary Joseph was the first to note the link between umbilical nodules and intra abdominal malignancy [1-4].

### Epidemiology

The occurrence of cutaneous metastases from malignant neoplasms occurs in from 1% to up to 9% of individuals, as determined at autopsy. Those metastases to the umbilicus are uncommon and represent only 10% of all secondary tumours which have spread to the skin [5,6]. Epidemiological studies showed that this condition predominates in females [7].

From a review of the literature, umbilical neoplastic nodules can be due to a primary tumour in 38% of cases, due to endometriosis in 32% of individuals, and in 30% are actually secondary tumour deposits from a primary tumour elsewhere [8]. If these nodules are secondary tumour deposits then the source of the primary tumour may be from the gastrointestinal (35–65%) and genitourinary (12–35%) tract. In addition, in 3–6% of cases it originates from haematological malignancies, or lung and breast cancers. In 15% to 30% of patients the source of the primary site of the tumour remains unknown [9-11].

### Clinical findings

Sister Mary Joseph's nodules usually present as a painful lump on the anterior abdominal wall. It has irregular margins and a hard fibrous consistency. The surface may be ulcerated and necrotic, with either blood, serous, purulent, or mucous discharge from it. The size of the nodule usually ranges from 0.5 to 2 cm, although some nodules may reach up to 10 cm in size [8]. High-resolution ultrasound (US) helps to clarify the clinical findings by detecting solid umbilical nodules, even if the diagnosis is difficult to make clinically. Moreover, careful examination and imaging of the abdominal contents may also point to the diagnosis [12].

### Pathophysiology

A full understanding of the mechanisms whereby the tumour spreads to the umbilicus remains unclear. However, following anatomical criteria, and several hypothesis have been proposed.

The umbilical ring is a scar invaginated on the abdominal wall between the transversalis fascia and peritoneum. After birth, the foetal cord structures develop into ligaments or peritoneal folds: 1) median umbilical ligament secondary to the obliterating urachus, 2) medial umbilical ligaments (which are obliterated umbilical arteries); 3) ligamentum teres (obliterated left umbilical vein) that continues into 4) the falciform ligament. On the lateral umbilical folds the inferior epigastric vessels and, sometimes, a vestigial vitelline duct connecting the umbilicus to the ileum can be recognised. The umbilical region shows a rich arterial supply that includes the inferior epigastric and deep circumflex iliac branches of the external iliac artery, and the superior epigastric branch of the internal mammary artery.

The venous drainage includes several anastomotic branches, coming from cranially the axillary vein, through the internal mammary vein, and caudally, the femoral vein through the superficial epigastric vein. In addition, the umbilicus may be connected with the portal system, through small umbilical veins.

The lymphatic system connects the umbilical region to the axillary, inguinal, and para-aortic lymph nodes. The deep lymphatic system passes along the falciform ligament, pierces the diaphragm and enters the anterior mediastinum or courses to the nodes around the iliac arteries [12-15].

All these systems (arterial, venous and lymphatic) as described represent possible routes by which metastatic tumour cells could implant into the umbilical region.

It is reasonable to suggest that direct extension of tumour through the peritoneum is the preferred route for gastrointestinal tumours. Furthermore, the common association between hepatic and umbilical metastases might suggests that the hypothesis that the tumour spreads from the primary tumour to the liver through the portal system and then through the lymphatic and/or venous channels, they spread to the umbilicus. It is still unclear if the umbilical tumour spread precedes the hepatic spread or *vice versa*.

Renal cell carcinoma typically spreads via extra-renal extension, lymphatic dissemination or venous invasion by the tumour. Intraperitoneal spread may occur as a result of disruption of the renal capsule [16]. The dissemination of neoplastic cell through the urachus is assumed to be the mechanism for the bladder cancers.

Haematogenous, lymphatic and venous spread all represent valid mechanisms of tumour spread from gynaecological cancers [7,14].

### Prognosis and therapy

Usually the presence of an umbilical metastasis indicates a poor prognosis, is a sign of advanced neoplastic disease, and may not be amenable to surgery. The survival of these patients without treatment has been reported to range from 2 to 11 months from the time of initial diagnosis [17-19].

However, recent studies have suggested that there are several factors which are able to influence the prognosis of such patients. Certain data has shown a better survival (mean 9.7 months) in patients who detect an umbilical metastasis before definitive treatment of the primary tumour. In contrast, when the lesion appears after the primary tumour has been treated then the survival for these patients does not exceed the 7.6 months [16,17].

Moreover, the aetiology of the primary malignancy determines the prognosis. For example, a better survival rate for patients with primary ovarian carcinoma has been reported previously [14].

Finally, the type of treatment seems able to influence the patient's prognosis. Despite some authors proposing only palliative treatment because of these patients poor prognosis [10,17,20], recent studies have demonstrated that there is a better survival (21 months) for patients if they are treated with a combination of surgery and adjuvant therapy instead of surgery alone (7.4 months) or chemotherapy alone (10.3 months) [9,11,14,18].

Obviously, the appropriateness of such an aggressive treatment approach is determined by the clinical state of the patient.

## Conclusion

A careful examination of all umbilical lesions is recommended, especially in those patients with gastrointestinal and genitourinary tract malignancies. All umbilical mass lesions should be biopsied to determinate the pathological nature of the lesion.

Clinical experience suggests that, whenever it is possible, an aggressive surgical approach combined with chemotherapy treatment may be considered to offer the patient the best survival probability.

## Competing interests

The author(s) declare that they have no competing interests.

## Authors' contributions

**RG **contributed to conception, design, literature search and preparation of manuscript.

**MC **contributed to conception, design, literature search and preparation of manuscript.

**FE **contributed to conception, design, literature search and preparation of manuscript.

**MB **contributed to conception, design, literature search and preparation of manuscript.

All authors read and approved the manuscript
